# Identification of inflammatory subgroups of schizophrenia and bipolar disorder patients with HERV-W ENV antigenemia by unsupervised cluster analysis

**DOI:** 10.1038/s41398-021-01499-0

**Published:** 2021-07-06

**Authors:** Ryad Tamouza, Urs Meyer, Marianne Foiselle, Jean-Romain Richard, Ching-Lien Wu, Wahid Boukouaci, Philippe Le Corvoisier, Caroline Barrau, Alexandre Lucas, Hervé Perron, Marion Leboyer

**Affiliations:** 1 AP-HP, Hôpital Henri Mondor, Département Médico-Universitaire de Psychiatrie et d’Addictologie (DMU IMPACT), Fédération Hospitalo-Universitaire de Médecine de Précision (FHU ADAPT), Créteil, France; 2 Université Paris Est Créteil, Laboratoire Neuro-Psychiatrie translationnelle, Créteil, France; 3 Fondation FondaMental, Créteil, France; 4 Institute of Pharmacology and Toxicology, University of Zurich-Vetsuisse, Zurich, Switzerland; 5 Neuroscience Center Zurich, University of Zurich and ETH Zurich, Zurich, Switzerland; 6 Université Paris Est Créteil, Centre Investigation Clinique, CIC Henri Mondor, Créteil, France; 7 Plateforme de Ressources Biologiques, HU Henri Mondor, Créteil, France; 8 Institut des Maladies Métaboliques et Cardiovasculaires (I2MC), plateau We-Met, Inserm UMR1048 and Université Paul Sabatier, Toulouse, France; 9 GeNeuro, 3, Chemin du pré Fleuri 1228 Plan-les-Ouates, Geneva, Switzerland; 10 Université de Lyon-UCBL, Lyon, France

**Keywords:** Predictive markers, Molecular neuroscience

## Abstract

Human endogenous retroviruses (HERVs) are remnants of infections that took place several million years ago and represent around 8% of the human genome. Despite evidence implicating increased expression of HERV type W envelope (HERV-W ENV) in schizophrenia and bipolar disorder, it remains unknown whether such expression is associated with distinct clinical or biological characteristics and symptoms. Accordingly, we performed unsupervised two-step clustering of a multivariate data set that included HERV-W ENV protein antigenemia, serum cytokine levels, childhood trauma scores, and clinical data of cohorts of patients with schizophrenia (*n* = 29), bipolar disorder (*n* = 43) and healthy controls (*n* = 32). We found that subsets of patients with schizophrenia (~41%) and bipolar disorder (~28%) show positive antigenemia for HERV-W ENV protein, whereas the large majority (96%) of controls was found to be negative for ENV protein. Unsupervised cluster analysis identified the presence of two main clusters of patients, which were best predicted by the presence or absence of HERV-W ENV protein. HERV-W expression was associated with increased serum levels of inflammatory cytokines and higher childhood maltreatment scores. Furthermore, patients with schizophrenia who were positive for HERV-W ENV protein showed more manic symptoms and higher daily chlorpromazine (CPZ) equivalents, whereas HERV-W ENV positive patients with bipolar disorder were found to have an earlier disease onset than those who were negative for HERV-W ENV protein. Taken together, our study suggest that HERV-W ENV protein antigenemia and cytokines can be used to stratify patients with major mood and psychotic disorders into subgroups with differing inflammatory and clinical profiles.

## Introduction

Within the context of precision medicine, the use of biological markers to reformulate/redefines complex diseases have revolutionized many medical fields, but, as of yet, classification and treatment of psychiatric disorders still rely on clinical symptomatology. Bipolar disorder (BD) and schizophrenia (SZ), the two major psychosis categories, overlap in symptoms, susceptibility genes and environmental risk factors, as well as with regards to therapeutic strategies [1]. “Psychosis” could be a final endpoint for multiple etiologies, while a useful complementary approach may include the identification of biological pathways enabling to identify homogeneous subgroups. The identification of biologically-based psychiatric diseases represents a considerable challenge for psychiatry today and solving it would represent a major step towards characterizing homogeneous subgroups.

Within the field of Immuno-Psychiatry [2], immune-related loci and mobile genetic elements are emerging as central players in the etiology of psychotic disorders [3–5]. Mobile genetic elements include the human endogenous retroviruses (HERVs), which are remnants of infections that took place million years ago and represent around 8% of the human genome [6]. Initially considered as “junk” DNA, endogenous retroviruses were found to control gene regulatory networks pertaining to human brain evolution and development [7–10] and are increasingly associated with brain disorders [3, 11–13]. The human genome harbors many distinct families of HERVs, including copies that can be transcribed under certain conditions [14]. Transcribable HERVs, including HERV-W, HERV-K, and HERV-H, are usually silenced by epigenetic machineries [15, 16], but can be activated/reactivated following infectious challenges and other pathological conditions of cellular stress [17, 18].

We and others have previously provided converging evidence of significant HERV-W envelope (ENV) protein expression, with elevated RNA transcription and variations of DNA copy numbers in psychotic disorders, including SZ and BD [3, 5, 19, 20]. More recently, we showed that HERV-W ENV protein can disrupt the central glutamatergic neurotransmitter system and cause psychosis-related behavioral impairments in murine models [21]. The latter experimental findings provided additional evidence for the hypothesis that increased HERV-W activity may be involved in the etiopathogenesis of psychotic disorders. It currently remains unknown, however, whether the expression of HERV-W is altered in only a subset of patients with psychotic disorders and/or whether altered HERV-W expression is associated with distinct clinical or biological characteristics and symptoms.

Therefore, we characterized HERV-W antigenemia for the envelope protein from the HERV-W family (HERV-W ENV) in a cohort of SZ and BD patients and healthy control (HC) subjects, for which detailed clinical annotations along with multiple serum biomarkers were available for unsupervised cluster analyses. With regard to serum biomarkers, we included an array of inflammatory cytokines (interleukin [IL]-1β, IL-4, IL-6, IL-8, tumor necrosis factor [TNF]-α, and interferon [IFN]-γ), which were previously found to be altered in (a subset of) SZ and/or BD patients [22–26]. All patients were evaluated for mania, depression, as well as for positive and negative psychotic symptoms. Our cluster analysis further incorporated the history of childhood maltreatment, which in turn has previously been found to increase inflammatory cytokines and other markers of inflammation in adulthood [24, 27–29].

## Patients and methods

### Participants

Patients with SZ or BD were systematically assessed and recruited in the university affiliated psychiatric department of Mondor Hospital (Créteil, France) between 2013 and 2019 under the framework of the French National granted I-GIVE (Immuno-Genetics, Inflammation, retro-Virus, Environment) cohort. They were included either during an acute episode of their disease i.e. BD (manic/hypomanic or depressive) or SZ, or as outpatients assessed for a standardized workup in the BD or SZ expert centers [30, 31]. Healthy controls (HC) were recruited in the Clinical Investigation Center (CIC) of Henry Mondor Hospital (Créteil, France).

Patient inclusion criteria were as follows: age above 18 years, absence of pregnancy or breastfeeding, absence of infectious event or vaccination within the preceding 4 weeks, negative serology for human immunodeficiency viruses (HIV1 + 2), Hepatitis A, B, and C viruses, and no reported inflammatory, auto-immune or neurological disorder. For HC: absence of any somatic disease, absence of any personal or familial history of psychiatric disorder. All subjects gave written informed consent for their participation in the study, which was approved by the Comité de Protection des Personnes Ile-de-France III.

### Clinical assessment

Patient diagnosis was established using the French version of the Structured Clinical Interview for DSM-IV [32] while HC were assessed using the French version of the Diagnostic Interview for Genetic Studies (DIGS) [33]. Mania and depression were evaluated using the Young Mania Rating Scale (YMRS) [34]. Depression was also scored using the Calgary Depression Scale (CDSS) [35] and the Montgomery-Asberg Depression Rating Scale (MADRS) [36], for SZ and BD respectively, whereas psychotic symptoms were assessed using the Positive and negative syndrome scale (PANSS) [37]. Subjects who scored MADRS below 15, YMRS below 8, and PANSS below 60 were considered in a stable phase. Stable, non-symptomatic patients and healthy controls were evaluated once, whereas patients hospitalized for an acute episode were assessed at admission and before discharge. Age of onset, chlorpromazine equivalents, and body mass index (BMI) were recorded. Blood samples were collected upon clinical evaluation and immediately sent to the Biological Research repository of the Henry Mondor University Hospital for processing and storage under adequate conditions. All participants were carefully interviewed by trained psychiatrists or psychologists. For further experiments, all samples were registered with a code and the experiments were performed without knowledge of the diagnostic and clinical status, or of the apparently healthy condition. Samples were unblinded by the principal investigator who communicated the correspondence of clinical data to the statistician who had separately receive the results of analyses with the codes only. Codes were broken by merging the two lists for cluster analyses.

### Detection of HERV-W envelope protein

For the detection of HERV-W ENV antigen in sera and the quantification of its circulating soluble form, all analyses were performed according to the conditions provided in the patent published under ref. WO2019201908 (A1) and entitled “Method for the detection of the soluble hydrophilic oligomeric form of HERV-W envelope protein”. Samples stored in freezers for more than a year, already thawed after initial freezing, not aliquoted and frozen from fresh serum were excluded while samples in tubes aliquoted and frozen once only from fresh serum; stored at −80 °C for a period less than one year before protein extraction for immunocapillary western blot analysis were maintained for further quantification. Detailed description of HERV-W ENV antigen detection is provided in Supplementary Information.

### Measurement of serum cytokines

Circulating serum levels of IL-1β, IL-4, IL-6, IL-8, TNF-α, and IFN-γ were quantified as described in Supplementary information.

### Assessment of childhood trauma scores

History of childhood maltreatment was recorded using the Childhood Trauma Questionnaire (CTQ) [38], a retrospective self-rating scale evaluating five types of maltreatment: emotional abuse (EA), emotional neglect (EN), physical abuse (PA), physical neglect (PN) and sexual abuse (SA) from age 0 to 18. The five dimensions are quoted on a 5-points Likert scale from “Never” to “Very Often”. Scores for each subtype of maltreatment were recorded for each subject. Psychometric qualities of CTQ have been previously demonstrated [39]. CTQ has been applied for patients and HC.

### Statistical analyses

A detailed description of the statistical tests used is provided in the Supplementary Information. All statistical analyses were performed using SPSS Statistics (version 25.0, IBM, Armonk, NY, USA) and Prism (version 8.0; GraphPad Software, La Jolla, California), with statistical significance set at *p* < 0.05.

## Results

### Demographics

The selected study cohort corresponding to the samples stored at −80 °C for less than a year and to never thawed tubes according to HERV-W ENV detectability conditions, consisted of 43 BD, 29 SZ patients and 32 HC (*n* = 104). The demographic characteristics of SZ, BD, and HC subjects are summarized in Table 1. When considering SZ and BD patients as distinct diagnostic entities, we observed that patients significantly differed from HC in terms of: (i) age (BD: 40.7 years (y) ± 17.30; SZ: 34.17 y ± 11.75; HC: 28.03 y ± 11.05; *p* = 0.0007); (ii) gender (BD: 57% of females; SZ: 31%; HC: 39%; *p* = 0.0007) and (iii) BMI (BD: 27.72 ± 6,87, SZ: 26.32 ± 6.87, HC: 20.32 ± 6.07; *p* < 0.0001). In terms of childhood maltreatment, BD and SZ exhibited significantly more emotional abuse than controls (BD: 9.45 ± 5.06; SZ: 10.9 ± 5.05; HC: 6.71 ± 2.97, *p* = 0.0015), but only BD subjects reported more emotional neglect (BD: 12.97 ± 5.97; HC: 8.33 ± 3.54; *p* = 0.0042) and sexual abuse (BD: 7.16 ± 3.92, HC: 5 ± 0, *p* = 0.0035) than HC subjects. Regarding clinical characteristics among patients groups, SZ subjects showed a significantly higher PANSS score than BD (SZ: 71.29 ± 20,83, BD: 59.2 ± 19.68, *p* = 0,0192). There were no significant differences in the age of onset, MADRS and YMRS score, and chlorpromazine (CPZ) equivalents between SZ and BD when SZ and B patients were considered as distinct diagnoses.

**Table 1:** Demographic characteristics of the study sample.

Variable	BP (*n* = 43)	SCZ (*n* = 29)	HC (*n* = 32)	Statistics	
Age^a,b^	40,70 (17,3)	34,17 (11,75)	28,03 (11,05)	*p* = 0,0007	BP > HC < SCZ
Sex^c,d^	56,82	31,03	39,39	*p* = 0,0007	
Age of onset^a,e^	23,57 (9,14)	20,48 (3,90)	—	ns	
MADRS^a,e^	14,73 (9,52)	14,35 (10,84)	—	ns	
YMRS^a,e^	15,03 (12,65)	12,48 (8,46)	—	ns	
PANSS^a,e^	59,2 (19,68)	71,29 (20,83)	—	*p* = 0,0192	
Chlorpromazine^a,e^	300,78 (265,8)	343,75 (209,66)	—	ns	
CTQ^a,b^	44,58 (15,82)	41,74 (11,51)	32,61 (8,67)	*p* = 0,0019	BP > HC
Physical abuse^a,b^	6,58 (2,73)	6,57 (2,69)	5,9 (1,9)	ns	
Sexual abuse^a,b^	7,16 (3,92)	5,7 (1,84)	5 (0)	*p* = 0,0035	BP > HC
Emotional abuse^a,b^	9,45 (5,06)	10,9 (5,05)	6,71 (2,97)	*p* = 0,0015	BP > HC < SCZ
Physical neglect^a,b^	7,79 (3,23)	8,14 (2,52)	6,63 (2,36)	ns	
Emotional neglect^a,b^	12,97 (5,97)	10,6 (4,79)	8,33 (3,54)	*p* = 0,0042	BP > HC
BMI^a,b^	27,72 (6,87)	26,32 (6,87)	20,32 (6,07)	*p* < 0,0001	BP > HC < SCZ

^a^Mean (SD).

^b^Kruskall Wallis’s test and Tukey’s test.

^c^% of female.

^d^Chi square’s test.

^e^Mann-Whitney’s test.

### Circulating serum levels of HERV-W envelope protein

**Fig. 1:**
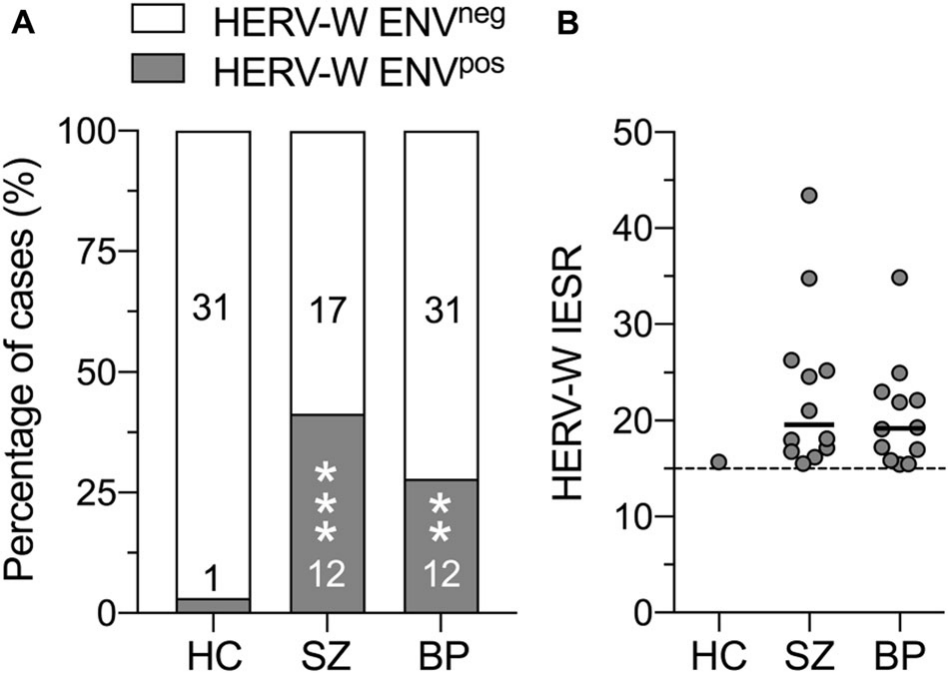
HERV-W envelope protein antigenemia in healthy controls (HC), patients with schizophrenia (SZ) and patients with bipolar disorder (BP). **A** Relative frequency distribution (depicted as percentage of cases) of HC, SZ and BP subjects who are negative (HERV-W ENV^neg^) or positive (HERV-W ENV^pos^) for HERV-W envelope protein. The embedded numbers reflect the number of subjects in each category. **p < 0.01 and ***p < 0.001, based on Chi-square test. **B** Quantity of HERV-W ENV soluble antigen in sera of HERV-W ENV^pos^ HC, SZ and BP subjects, expressed as “inter-experiment standardized result” (IESR). The dashed line indicates the cut-off value of 15 IESR. Values above and below this threshold were considered as positive and negative, respectively.

We compared the circulating serum levels of HERV-W ENV protein between HC, SZ and BD subjects. As graphically depicted in Fig. 1A, more than 96% (31 out of 32) of the HC subjects were negative for HERV-W ENV protein (HERV-W ENV^neg^), while only 1 out of 32 HC subjects was positive (HERV-W ENV^pos^). By contrast, 41.4% (12 out of 29) and 58.6% (17 out of 29) of the SZ cases were HERV-W ENV^pos^ and HERV-W ENV^neg^, respectively (Fig. 1A), leading to a significant difference in the number of cases with detectable HERV-W ENV protein levels between HC and SZ subjects (χ^2^ = 13.28, *z* = 3.64, *p* < 0.001). Likewise, there was a significant difference in the number of cases with detectable HERV-W ENV protein between HC and BD subjects (χ^2^ = 7.86, *z* = 2.80, *p* < 0.01). In the latter group, 27.9% (12 out of 43) of BD cases were HERV-W ENV^pos^, whereas 72.1% (31 out of 43) of BP cases were HERV-W ENV^neg^ (Fig. 1A). In HERV-W ENV^pos^ SZ and BD cases, the IESR index of HERV-W ENV protein ranged from 15.4 to 43.4, whereas the only one identified HERV-W^pos^ HC subject had an IESR index of 15.7 (Fig. 1B).

### Identification of patient subgroups

**Fig. 2:**
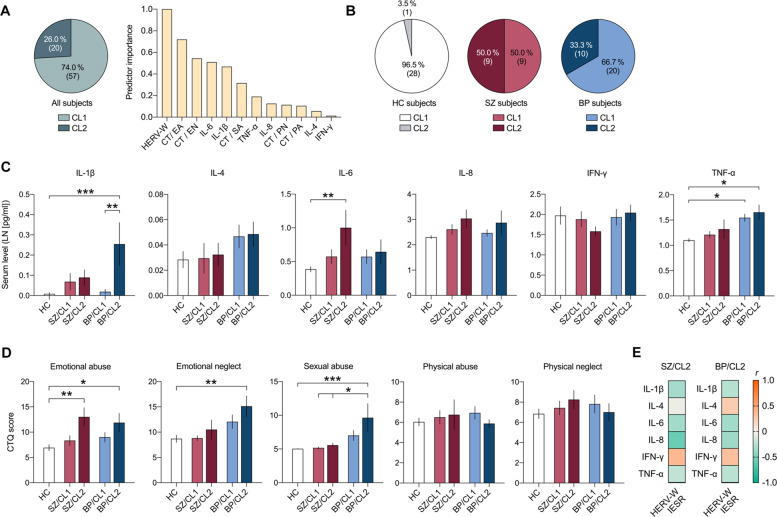
Stratification of healthy controls (HC), patients with schizophrenia (SZ) and patients with bipolar disorder (BP) based on two-step cluster analysis of HERV-W envelope protein antigenemia, serum cytokines (IL-1β, IL-4, IL-6, IL-8, TNF-α, IFN-γ) and childhood trauma (CT) questionnaire scores. The latter included scores on emotional abuse (EA), emotional neglect (EN), sexual abuse (SA), physical abuse (PA), and physical neglect (PN). **A** The pie chart shows the cluster distribution of all subjects (i.e., the entire sample of HC, SZ and BP subjects) across the two clusters (CL1 and CL2) identified by two-step cluster analysis. The numbers in brackets represent the number of subjects in each cluster. The bar plot shows the relative predictor importance for cluster separation as revealed by two-step cluster analysis. **B** The pie charts show the cluster distribution (CL1 or CL2) separately for of HC, SZ and BP subjects. The numbers in brackets represent the number of subjects in each cluster. **C** Serum cytokine levels (LN-transformed; means ± S.E.M) in different subgroups of patients (SZ/CL1, SZ/CL2, BP/CL1 and BP/C2) and HC subjects. *p < 0.05, **p < 0.01 and ***p < 0.001, based on Tukey’s post-hoc tests for multiple comparisons following one-way ANOVA. **D** CT scores (means ± S.E.M) in different subgroups of patients (SZ/CL1, SZ/CL2, BP/CL1 and BP/C2) and HC subjects. *p < 0.05, **p < 0.01 and ***p < 0.001, based on Tukey’s *p*ost-hoc tests for multiple comparisons following one-way ANOVA. **E** The heat map represents Pearson correlation coefficient (*r*) values for correlations between HERV-W IESR and different cytokines in the HERV-W ENV^pos^ SZ (SZ/CL2) and BP (BP/CL2) subgroups. None of the correlations attained statistical significance.

We performed a two-step cluster analysis to identify possible subgroups, thereby concomitantly integrating the available measures of HERV-W ENV positivity (CO: IESR > 15), serum cytokine levels (IL-1β, IL-4, IL-6, IL-8, TNF-α, IFN-γ) and CT scores (EA, EN, PA, PN, SA scores) from HC, SZ and BD subjects. The cluster analysis identified two main clusters (CL1 and CL2) with good cluster separation (silhouette measure of cohesion and separation > 0.6). As shown in Fig. 2A, 74.0% (57 out of 77) of all study subjects were identified as belonging to CL1, whereas 26.0% (20 out of 77) classified into CL2. Positivity for HERV-W ENV protein had the strongest predictor importance for cluster separation, followed by EA scores, EN scores, serum IL-6 levels and serum IL-1β levels (Fig. 2A). On the other hand, serum levels of IFN-γ had the lowest predictor importance for cluster separation (Fig. 2A). Based on the strong predictor importance of HERV-W ENV positivity, all subjects who were HERV-W ENV^pos^ were assigned to CL2, whereas all HERV-W ENV^neg^ were classified as belonging to CL1. Figure 2B graphically depicts the relative distribution of HC, SZ and BD subjects according to clusters. Notably, only 3.5% (1 out of 29) of HC subjects were identified as belonging to CL2, 50.0% (9 out of 18) and 33.3% of (10 out of 30) of SZ and BD patients, respectively, were assigned to this cluster (Fig. 2B).

Following stratification of the study sample into subgroups, we compared the serum cytokine levels between the different subgroups of patients (SZ/CL1, SZ/CL2, BD/CL1 and BD/CL2) and HC subjects. This analysis revealed significant subgroup-specific effects for IL-1β (ANOVA: *F*_(4,71)_ = 4.72; *p* < 0.01) and IL-6 (ANOVA: *F*_(4,71)_ = 3.47; *p* < 0.01). Tukey’s post-hoc tests for multiple comparisons showed that serum IL-1β levels were elevated specifically in HERV-W ENV^pos^ BD subjects (i.e. in the BD/CL2 subgroup), as compared to HERV-W ENV^neg^ BD (BD/CL1) subjects (*p* < 0.01) and HC subjects (*p* < 0.001; Fig. 2C). On the other hand, serum IL-6 levels were elevated significantly in HERV-W ENV^pos^ SZ subjects (i.e. in the SZ/CL2 subgroup), as compared to HC subjects (*p* < 0.01; Fig. 2C). The serum levels of TNF-α were elevated in BD subjects regardless of whether they were positive or negative for HERV-W ENV protein (Fig. 2C), as supported by ANOVA (*F*_(4,71)_ = 4.50; *p* < 0.01) and subsequent post-hoc comparisons revealed significant differences between HC subjects and BD/CL1 (*p* < 0.05) or BD/CL2 (*p* < 0.05) subjects. By contrast, the serum levels of IL-4, IL-8 and IFN-γ were not significantly different between diagnoses and/or patient subgroups (Fig. 2C). Furthermore, none of the correlations between IESR of HERV-W ENV protein and serum cytokines attained statistical significance, neither in the CL2/SZ nor the CL2/BP subgroup (Fig. 2C).

We further compared the different subgroups of patients (SZ/CL1, SZ/CL2, BD/CL1 and BD/C2) and HC subjects in terms of CT scores. We revealed subgroup-specific effects for EA (*F*_(4,60)_ = 5.35; *p* < 0.01), EN (*F*_(4,60)_ = 3.65; *p* < 0.05) and SA (*F*_(4,60)_ = 5.459; *p* < 0.001) scores. These CT scores were significantly higher in HERV-W ENV^pos^ BD subjects (i.e. in the BD/CL2 subgroup) as compared to HC subjects (EA: p < 0.05; EN: *p* < 0.01; SA: *p* < 0.001), whereas they were not significantly different in HERV-W ENV^neg^ BD subjects (Fig. 2D). Likewise, only HERV-W ENV^pos^ but not subjects HERV-W ENV^neg^ SZ subjects displayed a significant (p < 0.01) increase in EA scores compared with HC subjects (Fig. 2D). On the other hand, PA and PN scores did not differ significantly between diagnoses and/or patient subgroups (Fig. 2D).

### Clinical characteristics of patient subgroups

In a next step, we examined whether the two subgroups of SZ and BD patients i.e. CL1 and CL2, also differ in terms of clinical characteristics. Using the subgroup identification revealed by the preceding clusters analysis (Fig. 2), we compared the age of the disease onset (defined as the age at which the first episode of psychiatric illness occurred), MADRS score, YMRS score, PANSS scores, and daily CPZ equivalents between the different subgroups of patients (SZ/CL1, SZ/CL2, BD/CL1, and BD/C2) and, whenever possible, HC subjects. Daily CPZ equivalents were analyzed for SZ patients only.

**Fig. 3:**
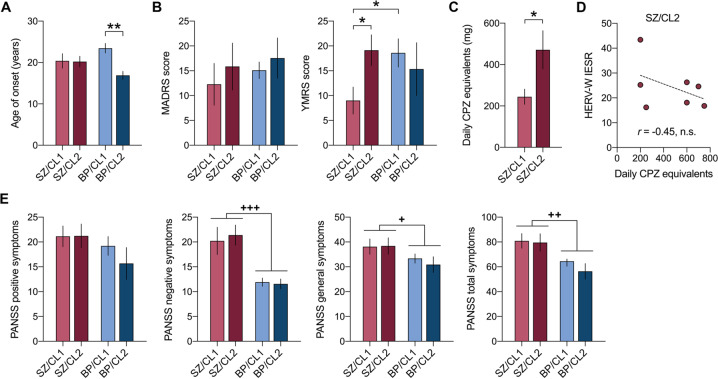
Clinical characteristics of different subgroups of patients with schizophrenia (SZ) and bipolar disorder (BP). The subgroups were defined by a preceding two-step cluster analysis (see Fig. 2), revealing two main clusters (CL1 and CL2) in each diagnostic group. **A** Age of the disease onset (in years), defined as the age at which the first episode of psychiatric illness occurred. **B** Montgomery-Asberg Depression Rating Scale (MADRS) and Young Mania Rating Scale (YMRS) scores. **C** Daily intake (mg) of chlorpromazine (CPZ) equivalents (available for SZ patients only). **D** Correlation between HERV-W IESR and daily CPZ equivalents in the HERV-W ENV^pos^ SZ (SZ/CL2) subgroup. **E** Positive and Negative Syndrome Scale (PANSS) scores. All values represent means ± S.E.M. *p < 0.05 and **p < 0.05 based on Tukey’s post-hoc tests for multiple comparisons following two-way ANOVA; ^+^*p* < 0.05, ^++^*p* < 0.01 and ^+++^*p* < 0.001, reflecting significant main effects of diagnostic group (SZ or BP, irrespective of cluster association) in two-way ANOVA.

ANOVA of the age of the disease onset revealed a significant interaction between diagnosis and subgroups (*F*_(1,43)_ = 4.15; *p* < 0.05), indicating that the disease onset was influenced by both factors. As shown in Fig. 3A, HERV-W ENV^pos^ BD subjects had a significantly earlier disease onset than HERV-W ENV^neg^ BD subjects (mean ± S.E.M. = 17.9 ± 1.7 years vs mean ± S.E.M. = 23.1 ± 1.2 years, *p* < 0.01 respectively), whereas the disease onset of SZ patients did not differ as a function of subgroups (mean ± S.E.M. in SZ patients = 20.5 ± 0.8 years). In addition, there were subgroup-specific effects in the analysis of YMRS score, as indicated by the significant interaction between diagnosis and subgroups (*F*_(1,40)_ = 6.12; *p* < 0.05). As shown in Fig. 3B, the YMRS scores were significantly (*p* < 0.05) higher in HERV-W ENV^pos^ SZ subjects than in HERV-W ENV^neg^ SZ subjects and were comparable to those measured in HERV-W ENV^neg^ or HERV-W ENV^pos^ BD subjects. By contrast, there were no subgroup-specific effects in terms of MADRS (Fig. 3B) or PANNS (Fig. 3D) scores. Compared to overall BD subjects, (HERV-W ENV^neg^ or HERV-W ENV^pos^), SZ patients generally scored higher on the PANNS negative symptoms (ANOVA, main effect of diagnosis: *F*_(1,42)_ = 30.09; *p* < 0.001), general symptoms (ANOVA, main effect of diagnosis: *F*_(1,42)_ = 4.71; *p* < 0.05) and total symptoms (ANOVA, main effect of diagnosis: *F*_(1,42)_ = 11.36; *p* < 0.01) scales (Fig. 3E).

We further found that the daily CPZ equivalents were significantly (*t* = 2.49, *p* < 0.05) increased in HERV-W ENV^pos^ SZ subjects (i.e. in the SZ/CL2 group) compared to HERV-W ENV^neg^ SZ subjects (i.e. in the SZ/CL1 group; Fig. 3C). There was, however, no significant correlation between daily CPZ equivalents and the IESR index of HERV-W ENV protein in HERV-W ENV^pos^ SZ subjects (Fig. 3D).

## Discussion

The findings from the present study provides additional evidence supporting the involvement of altered HERV-W activity in psychotic disorders. Using a capillary-based western blot method to measure HERV-W ENV antigen in sera [18], we replicated previous findings of significantly positive HERV-W ENV antigenemia in SZ subjects relative to controls [20]. Moreover, our study corroborates previous findings of increased HERV-W ENV RNA expression in peripheral blood mononuclear cells of BP subjects [3]. The present data, however, provide important extensions to these earlier findings. Firstly, we demonstrated that only a subset of SZ (~41%) and BD (~28%) patients showed positive antigenemia for HERV-W ENV protein, whereas the large majority (96%) of HC subjects was found to be negative for HERV-W ENV protein in sera. Secondly, the use of unsupervised cluster analysis identified the presence of two main clusters of patients, which were best predicted by the presence (cluster 2) or absence (cluster 1) of HERV-W ENV protein. Importantly, the cluster analysis further demonstrated that HERV-W expression is associated with distinct biological and clinical features in SZ and BD patients. More specifically, HERV-W ENV^pos^ SZ and BD subjects displayed increased serum levels of inflammatory cytokines and higher childhood maltreatment scores as compared to HERV-W ENV^neg^ SZ and BD subjects. Furthermore, HERV-W ENV^pos^ SZ subjects showed more manic symptoms and higher daily CPZ equivalents than HERV-W ENV^neg^ SZ subjects, whereas HERV-W ENV^pos^ BD subjects had an earlier disease onset than HERV-W ENV^neg^ BD subjects. The latter observation may be indicative of an early immunological trigger, given that elevations in circulating inflammatory markers and inflammation-related cardiovascular abnormalities have been repeatedly found in young adults with BD [40, 41]. Similarly, the association between HERV-W ENV positivity and elevated score of mania in SZ patients could involve inflammatory processes, as previously suggested by others [42]. Taken together, our data support the current hypothesis that differences in immune-related biological factors may contribute to the clinical heterogeneity in SZ and BD [43]. Furthermore, if replicated and extended in future studies, our findings may support the use of HERV-W ENV protein antigenemia as a biomarker to stratify SZ and/or BD patients into subgroups with differing clinical manifestations and needs for more tailored treatment.

Our clustering approach was based on unsupervised two-step clustering, which is capable of concomitantly integrating continuous variables (such as serum cytokine levels) and categorical variables (such as positivity for HERV-W ENV protein). The identification of two main clusters in our data set is consistent with previous clustering approaches that aimed to stratify patients with psychotic disorders based on inflammatory cytokine profiles using composite scores analyses [44], regularized regression [45], k-means clustering [26, 46, 47], or through a combination of unsupervised exploratory factor analysis and hierarchical clustering [48]. Consistent with our findings, the majority of these previous studies demonstrated that inflammatory profiles in patients with psychotic disorders can be sub-grouped into two main clusters, namely “high inflammatory” and a “low inflammatory” subgroups. The consistency between our findings here and those reported before [26, 46, 47] raises the question whether differential HERV-W activity may have contributed to the segregation of “high inflammatory” and a “low inflammatory” subgroups in previous clustering analyses of inflammation-related markers as applied to patients with psychotic disorders.

While both SZ and BD subjects with positive HERV-W ENV protein antigenemia displayed increased serum levels of inflammatory cytokines, the specificity of these inflammatory proxies differed between HERV-W ENV^pos^ SZ and BD subjects. Indeed, HERV-W ENV^pos^ SZ but not BD subjects showed elevated serum IL-6 levels, whereas serum IL-1β levels were only increased in HERV-W ENV^pos^ BD but not SZ subjects. These findings are consistent with a recent cross-disorder cluster analysis [49], showing that increased serum IL-6 levels are more strongly associated with SZ than BD. On speculative grounds, the variations in cytokine profiles between HERV-W ENV^pos^ SZ and BD subjects may be related to differences in the immune-related genetic architecture of SZ and BD [50, 51]. However, even though SZ and BP patients could be stratified based on the presence of HERV-W ENV protein and differing cytokine status in serum, there appeared to be no linear relationship between the actual levels of ENV protein and serum cytokines, at least when considering the sampling and detection techniques described here. Future investigations are warranted to explore the possible relationship between HERV ENV protein and inflammatory markers in serum, preferably using a larger sample size and comparing different HERV-W ENV quantification methods.

An alternative (but not mutually exclusive) explanation for the different cytokine profiles observed in HERV-W ENV^pos^ SZ and BD subjects may be related to the distinct history in childhood maltreatment. Several meta-analysis [24, 52] reported that childhood maltreatment is associated with elevated levels of circulating CRP, IL-6 and TNF-α at adult age, both in psychiatric patients and unaffected subjects. In a prospective longitudinal cohort study, high sensitivity CRP (hsCRP) levels were found to be higher in depressed patients with a history of childhood maltreatment as compared to those without childhood trauma exposure [53], while elevated levels of IL-6 and/or TNF-α pro-inflammatory cytokines were observed in psychotic patients and in first-episode psychosis patients with history of childhood maltreatment [54, 55]. However, while childhood maltreatment is generally considered to cause low-grade inflammation persisting into adulthood [24], this effect appears to be influenced by the severity and/or specificity of the trauma [27–29]. In the present cohort of patients and controls, we found that HERV-W ENV^pos^ BD but not SZ subjects reported more emotional neglect and sexual abuse as compared to HERV-W ENV^neg^ BD and SZ subjects and controls, whereas emotional abuse was similarly increased in HERV-W ENV^pos^ BD and SZ subjects. However, whether exposure to emotional neglect and/or sexual abuse may explain the specific increase in serum IL-1β occurring in HERV-W ENV^pos^ BD needs to be investigated further in future studies.

Consistent with the concept of multiple-hit theories of disease pathogenesis, we previously reported additive effects between childhood sexual abuse and gene variants encoding the toll-like receptor 2 (TLR2), a pathogen recognition receptor pertaining to innate immunity, on disease onset of BD [56]. In view of the findings presented here, it is tempting to speculate that the earlier disease onset in HERV-W ENV^pos^ relative to HERV-W ENV^neg^ BD subjects may involve intricate interactions between the genetic background and exposure to childhood trauma [17]. Based on previous findings showing un-silencing of HERV-W upon infection, inflammation and/or trauma [57–59], we speculate that exposure to childhood trauma may be one of events triggering re-activation of HERV-W ENV protein expression, which in turn may maintain inflammatory responses in a chronic state [2, 12, 60–64]. The precise mechanisms, by which this reactivation occurs, remain elusive and warrant further investigation. On speculative grounds, however, it may involve modulation of the epigenetic co-repressor protein, tripartite protein 28 (TRIM 28) [65–67], which is a key factor for maintaining HERV activity in a silenced state. Consistent with this effect, a recent study showed that CRISPR/cas9-mediated knockout of TRIM 28 during murine brain development resulted in high expression of neuronal ERV in adult brains [66]. Based on these findings, it would be interesting to further explore whether exposure to childhood trauma or to other environmental factors implicated in psychotic disorders, such as maternal immune activation [68–70], might lead to positive HERV-W ENV protein antigenemia through modulation of TRIM 28 activity.

We acknowledge a number of limitations of our study. Firstly, our study was based on a relatively small sample size, and therefore, our results need to be replicated in larger cohort of psychotic patients and controls. Secondly, the majority of patients were exposed to medications at the time of sample collection. Hence, we cannot rule out whether parts of our data may have been influenced by the patients’ medication status. This limitation appears particularly relevant in view of the numerous findings showing significant effects of antipsychotic drugs on inflammatory cytokines [71, 72]. We did not, however, find any correlation between daily CPZ equivalents and the IESR index of HERV-W ENV protein in HERV ENV^pos^ SZ subjects, suggesting that HERV-W ENV protein antigenemia is not associated with (or even induced by) antipsychotic drug exposure [3]. Nevertheless, to further corroborate our findings, it would be highly warranted to assess HERV-W ENV protein antigenemia and its relationship with inflammatory and clinical profiles in drug-naive, first-episode psychotic patients.

In summary, the present study provides further support for the involvement of altered HERV-W activity in psychotic disorders and suggests that HERV-W ENV protein antigenemia along with IL-6 and IL-1β circulating level evaluations can be used to stratify patients into subgroups with differing inflammatory and clinical profiles. Our findings may be relevant for biomarker-guided personalized medicine and for the future development of novel therapeutic strategies that are based on neutralizing HERV-W ENV protein in inflammatory pathologies and beyond [73].

## Supplementary information

Supplementary Information
