# Correction: Identification of inflammatory subgroups of schizophrenia and bipolar disorder patients with HERV-W ENV antigenemia by unsupervised cluster analysis

**DOI:** 10.1038/s41398-021-01572-8

**Published:** 2021-09-01

**Authors:** Ryad Tamouza, Urs Meyer, Marianne Foiselle, Jean-Romain Richard, Ching-lien Wu, Wahid Boukouaci, Philippe Le Corvoisier, Caroline Barrau, Alexandre Lucas, Hervé Perron, Marion Leboyer

**Affiliations:** 1 AP-HP, Hôpital Henri Mondor, Département Médico-Universitaire de Psychiatrie et d’Addictologie (DMU IMPACT), Fédération Hospitalo-Universitaire de Médecine de Précision (FHU ADAPT), Créteil, France; 2 Université Paris Est Créteil, Laboratoire Neuro-Psychiatrie translationnelle, Créteil, France; 3 Fondation FondaMental, Créteil, France; 4 Institute of Pharmacology and Toxicology, University of Zurich-Vetsuisse, Zurich, Switzerland; 5 Neuroscience Center Zurich, University of Zurich and ETH Zurich, Zurich, Switzerland; 6 Université Paris Est Créteil, Centre Investigation Clinique, CIC Henri Mondor, Créteil, France; 7 Plateforme de Ressources Biologiques, HU Henri Mondor, Créteil, France; 8 Institut des Maladies Métaboliques et Cardiovasculaires (I2MC), plateau We-Met, Inserm UMR1048 and Université Paul Sabatier, Toulouse, France; 9 GeNeuro, 3, Chemin du pré Fleuri 1228 Plan-les-Ouates, Geneva, Switzerland; 10 Université de Lyon-UCBL, Lyon, France

**Keywords:** Predictive markers, Molecular neuroscience

Correction to: *Translational Psychiatry* 10.1038/s41398-021-01499-0, published online 6 July 2021

The original version of this article unfortunately contained a mistake in an author name. The correct name of Ching-lieng Lu is Ching-Lien Wu. The authors apologize for the mistake. The original article has been corrected.

