# Supplementary material for: Identification of inflammatory subgroups of schizophrenia and bipolar disorder patients with HERV-W ENV antigenemia by unsupervised cluster analysis
Source: Transl Psychiatry. 2021 Jul 6;11:377. doi: 10.1038/s41398-021-01499-0 (PMC8260666; doi:10.1038/s41398-021-01499-0)
Supplement: Supplementary file 1 — Supplementary Information [file 41398_2021_1499_MOESM1_ESM.docx]

**Supplementary Information**

***Detection of HERV-W envelope protein***

Briefly, sera stored less than a year at -80C from never-thawed aliquots were only included for stability issues of the target antigen after long-term storage and/or previous freez-thaw cycle. They were diluted ½ in extraction buffer, containing RIPA buffer (Sigma Aldrich) supplemented with 1% Fos cholin (Anatrace) and protease inhibitor cocktail (Roche). Lysates were incubated for 2h at 25°C with gentle agitation (120 rpm). Total protein amount from each fractions were evaluated using the “Protein Assay Reagent” kit (Pierce). Then, 500µL of protein extract were load in “AMICON Ultra-0.5 100K” device (Merck-Millipore). High molecular weight proteins (>100kDa) were purified / concentrated on column filter by centrifugation 30min, 14 000 x g. Next, 50µg of total protein extract were deglycosylated using “Protein Deglycosylation Kit” (Promega). Deglycosylation was performed according manufacturer instructions during 18h at 37°C.

Western blots were performed using WES, an automated capillary-based size sorting system (Proteinsimple). Deglycosylated protein lysate was mixed with fluorescent master mix and heated at 95°C for 5 minutes. The samples, blocking reagent, wash buffer, primary antibodies, streptavidin or secondary antibodies coupled with HRP, and chemiluminescent substrate were dispensed into designated wells in a manufacturer provided microplate. The plate was loaded into the instrument and protein was drawn into individual capillaries on a 25 capillary cassette (66-440kDa) provided by the manufacturer. Protein separation and immunodetection was performed automatically on the individual capillaries using default settings. Primary antibody, GN_mAb_Env01-biotin (20µg/mL) was raised against pHERV-W ENV protein produced by clones from MS retroviral particles. The concentration of the HERV-W ENV soluble antigen in sera was expressed as “inter-experiment standardized result” (IESR). The IESR corresponded to the area under the curve (AUC) of the specific HERV-W ENV soluble antigen peak calculated from the automated capillary-based immunoassay software Compass (Proteinsimple), normalized for inter-experiment variations using the mean + 2 SDs of series of healthy controls (lower limit of specificity/positivity cut-off value: CO) in each experiment. This normalization served to adjust each sample to the reference data obtained from the first series of HERV-W ENV antigen detection, such that the measured AUC of each sample was multiplied by the ratio of corresponding CO. Thus, the normalization adjusted all values to the same mean of non-specific background signal. Specifically, all data measurements were standardized with a CO of 15 AUC. Sample values that were above this threshold were considered as positive for HERV-W (HERV-W^pos^). On the other hand, sample values that fell below the CO of 15 corresponded to non-specific background signal generated by the components of the sample and by the technical protocol with non-significant variations among negative samples (“technical background noise”), and were therefore considered as negative for HERV-W (HERV-W^neg^).

***Measurement of serum cytokines***

Circulating serum levels of IL-1β, IL-4, IL-6, IL-8, TNF-α, and IFN-γ were quantified using a Meso-Scale Discovery (MSD) human V-Plex electrochemoluminescence assay (MSD, Rockville, Maryland, USA) according to the manufacturer’s recommendations. The plates were analyzed using MSD’s Workbench analyzer and software package according to the manufacturer’s recommendations. All assays were run in duplicates according to the manufacturer’s instructions. The detection limits were as follows: IL-1β: 0.05–375 pg/mL, IL-4: 0.02–158 pg/mL, IL-6: 0.06–488 pg/mL, IL-8: 0.07–375 pg/mL, TNF-α: 0.04–248 pg/mL and IFN-γ: 0.2-938 pg/mL.

***Statistical analyses***

Demographic characteristics of study participants and HERV-W antigenemia were analyzed by chi-square tests. The sample size was chosen according to previous pilot studies that indicated that about one-half of patients with SZ had positive antigenemia for HERV-W ENV (1).Two-step cluster analysis was used to identify possible subgroups of patients and controls with differing HERV-W positivity, serum cytokines and/or CT scores. The two‐step cluster method uses a pre‐clustering step by scanning the entire dataset and storing the dense regions of data records in terms of summary statistics, after which a hierarchical clustering algorithm is then applied to the cluster dense regions (2,3). Hence, it represents a hybrid approach, which first uses a distance measure to separate groups and then a probabilistic approach to choose the optimal subgroup model. The two-step cluster method was chosen for two main reasons. First, it is capable of handling data sets that are composed of a mixture of categorical (e.g. HERV-W positivity) and continuous (e.g. serum cytokines and CT scores) variables (2,3). Second, it can be run without predetermining the number of clusters, thereby avoiding bias in terms of identifying the number of possible clusters (2,3). For data clustering, we concomitantly integrated measures of HERV-W positivity (CO: IESR > 15; categorical variable), serum cytokines (IL-1β, IL-4, IL-6, IL-8, TNF-α, IFN-γ; continuous variables) and CT scores (EA, EN, PA, PN, SA scores; continuous variables) from HC, SZ and BP subjects. Serum cytokines were first subjected to natural logarithmic transformation (LN) in order to minimize data skewness. Missing variables for some of the subjects led to a reduction in sample size, such that the final number of subjects included in the cluster analysis was: *n*(HC) = 29, *n*(SZ) = 18 and *n*(BP) = 30. Bayesian Criterion (BIC) was used to estimate of the maximum number of clusters, whereas the log­likelihood method was used as distance measure (2-4).

1. Perron, H. et al. Endogenous retrovirus type W GAG and envelope protein antigenemia in serum of schizophrenic patients. *Biol Psychiatry*, **64**,1019-23 (2008).

Gelbard, R., Goldman, O., Spiegler, I. Investigating diversity of clustering methods: an empirical comparison. *Data Knowl. Eng*, **63**, 155–166 (2007).

Kent, P., Jensen, R. K., Kongsted, A. A comparison of three clustering methods for finding subgroups in MRI, SMS or clinical data: SPSS twostep cluster analysis, latent Gold and SNOB. *BMC Med. Res. Methodol*, **14**:113 (2014).

1. Purves-Tyson, T.D. et al. Increased levels of midbrain immune-related transcripts in schizophrenia and in murine offspring after maternal immune activation. *Mol Psychiatry*, **10**.1038/s41380-019-0434-0 (2019).
